# iOS Appstore-Based Phone Apps for Diabetes Management: Potential for Use in Medication Adherence

**DOI:** 10.2196/diabetes.6468

**Published:** 2017-07-11

**Authors:** Mark Martinez, Su Bin Park, Isaac Maison, Vicky Mody, Lewis Sungkon Soh, Harish Singh Parihar

**Affiliations:** 1 Philadelphia College of Osteopathic Medicine - GA campus School of Pharmacy Suwanee, GA United States

**Keywords:** diabetes, telemedicine, blood glucose self-monitoring glucose monitoring, mobile applications, self-care, mobile health

## Abstract

**Background:**

Currently, various phone apps have been developed to assist patients. Many of these apps are developed to assist patients in the self-management of chronic diseases such as diabetes. It is essential to analyze these various apps to understand the key features that would potentially be instrumental in helping patients successfully achieve goals in disease self-management.

**Objective:**

The objective of this study was to conduct a review of all the available diabetes-related apps in the iOS App Store to evaluate which diabetic app is more interactive and offers a wide variety of operations such as monitoring glucose, water, carbohydrate intake, weight, body mass index (BMI), medication, blood pressure (BP) levels, reminders or push notifications, food database, charts, exercise management, email, sync between devices, syncing data directly to the prescribers, and other miscellaneous functions such as (Twitter integration, password protection, retina display, barcode scanner, apple watch functionality, and cloud syncing).

**Methods:**

Data was gathered using the iOS App Store on an iPad. The search term “diabetes” resulted in 1209 results. Many of the results obtained were remotely related to diabetes and focused mainly on diet, exercise, emergency services, refill reminders, providing general diabetes information, and other nontherapeutic options. We reviewed each app description and only included apps that were meant for tracking blood glucose levels. All data were obtained in one sitting by one person on the same device, as we found that carrying out the search at different times or on different devices (iPhones) resulted in varying results. Apps that did not have a feature for tracking glucose levels were excluded from the study.

**Results:**

The search resulted in 1209 results; 85 apps were retained based on the inclusion criteria mentioned above. All the apps were reviewed for average customer ratings, number of reviews, price, and functions. Of all the apps surveyed, 18 apps with the highest number of user ratings were used for in-depth analysis. Of these 18 apps, 50% (9/18) also had a medication adherence function. Our analysis revealed that the Diabetes logbook used by the mySugr app was one of the best; it differentiated itself by introducing fun as a method of increasing adherence.

**Conclusions:**

A large variation was seen in patient ratings of app features. Many patient reviewers desired simplicity of app functions. Glucose level tracking and email features potentially helped patients and health care providers manage the disease more efficiently. However, none of the apps could sync data directly to the prescribers. Additional features such as graph customization, availability of data backup, and recording previous entries were also requested by many users. Thus, the use of apps in disease management and patient and health-care provider involvement in future app refinement and development should be encouraged.

## Introduction

The Centers for Disease Control and Prevention define chronic diseases as “the most common, costly, and preventable of all health problems.” This includes conditions such as heart disease, stroke, and cancer [1]. In the United States, approximately 70% or 1.7 million of all deaths are due to chronic diseases [2].

Diabetes is a chronic disease that occurs in three primary forms: type 1 diabetes mellitus (T1DM), type 2 diabetes mellitus (T2DM), and gestational diabetes mellitus [3]. T1DM is characterized by pancreatic beta cells that are destroyed and thus cannot produce insulin [3]. T2DM is characterized by insulin resistance, markedly in muscles, liver, and adipose tissues [3]. Obesity [4,5], family history [6], physical inactivity [7], and ethnicity [8] are some of the risk factors associated with diabetes. The prevalence of diabetes in the United States in 2014 was 9.3% (29.1 million) and continues to rise [9]. Among adults aged 18 to 79 years, there were an estimated 1.4 million new cases of diabetes diagnosed in 2014 [10]. If this trend continues, as many as 1 out of every 3 adults in the United States could develop diabetes by 2050 [9].

Diabetes is managed through pharmacologic therapy and lifestyle modifications such as exercise, diet, and glucose monitoring [11], T1DM is defined by the patient’s inability to produce insulin, and thus, these patients are dependent on the administration of insulin for proper management of the disease. However, T2DM can be managed through the administration of oral hypoglycemic agents, insulin, non-insulin injectable agents, or a combination of agents [12]. Hemoglobin A1C and blood glucose values are used for diagnosis and management of diabetes [11]. Improper management of glycemic levels can lead to outcomes including, but not limited to, adverse cardiovascular outcomes, retinopathy, nephropathy, hyperglycemia, and hypoglycemia [13]. Nonadherence is one contributor to the improper management of diabetes as it is estimated that 50% of patients do not take their medications as prescribed by their physicians [14]. The New England Healthcare Institute estimates that nonadherence along with suboptimal prescribing, drug administration, and diagnosis could result in as much as US $290 billion per year in avoidable medical spending [15]. Therefore, compliance, or the proper use of medication by patients, plays a crucial role in the proper management of diabetes [16].

For patients diagnosed with diabetes, monitoring of blood glucose levels is the principal foundation of treatment planning and is performed through the use of a glucometer, a lancing device, and testing strips. Blood glucose values allow the patient or physician to adjust medication strength and also provide insight on disease progression. While A1C provides a 3-month average of blood glucose levels, it does not provide specific information that can support the adjustment of fasting plasma glucose or post-prandial glucose levels in the case of uncontrolled diabetes. An increased frequency of glucose level measurements has been associated with reduced Hemoglobin A1C levels [17]. Daily logging of self-monitored blood glucose levels may increase patient safety and awareness to therapeutic effectiveness as well as aid in adjustments to treatment planning.

With roughly half of the adult US population managing one chronic disease and 25% managing two or more chronic health conditions [1], it is becoming increasingly important to provide patients with the education and tools to self-manage their diseases. Nadkarni et al have shown that the implementation of a plan for self-monitoring behavior results in an increase in the frequency of blood glucose level measurements [18]. Moreover, it has been shown that rather than written documentation, electronic record-keeping may be of greater efficacy [19]. While mobile phones were once less accessible and desirable due to cost and limited functions, innovation has led to the mobile phone becoming ubiquitous throughout the United States and globally. Mobile phone ownership in 2015 among US adults ≥ 18 years old was an estimated 68% and 86% among those aged 18 to 29 years [20]. A variety of mobile phone apps tailored for the management of chronic diseases is available for download in the various app stores. Prior studies have shown that mobile phone app usage was correlated to patient behavioral patterns that facilitate diabetes self-management [21].

Prior studies have been conducted pertaining to a number of the apps included in this review [21,22,23]. However, some apps have been discontinued and many more have been added to the market since the publication of those studies. This review strives to provide a comprehensive analysis of currently available apps for tracking blood glucose levels.

The purpose of this article was to provide a qualitative review of the various apps related to diabetes self-management that are available on the iOS Apple App Store. A secondary objective was to provide a detailed analysis and comprehensive review of additional features with respect to the top 15 apps for tracking glucose levels, indicated by the number of reviews on the iOS App Store, in order to facilitate patient selection of apps.

## Methods

### Search Methodology

The search term used on the iOS App Store was “diabetes,” which was entered on an iPad, resulting in 1209 apps as of 6th September, 2015. The iPad was selected instead of the iPhone-6 because of increased screen space and reduced tendency to crash. The search term “diabetes” produced an excessive number of results that led to crashes on an iPhone-6 while browsing through the pages. For instance, if 100 pages of search results were produced, a crash was possible at any point when changing pages, at which point a new search would have to be performed, resulting in a different order of results and thus making it more difficult to extract information.

In addition, the iPad was selected instead of a desktop because the desktop version of the App Store does not display the total number of results. The iOS App Store was also preferred over the Play Store for Android as the Play Store tends to overestimate results through generation of less relevant results [24,25]. Moreover, the scope of this research was to only to review the apps available for iOS. Hence, Google’s Play Store was not reviewed. The broad search term “diabetes” was used to maximize the number of results pertaining to potential apps for tracking glucose levels.

**Table 1:** List of diabetes self-management apps available in iOS App Store.

No.	App name	Average customer rating	Number of reviews	Price (US $)	Functions (1^a^, 2^b^, 3^c^, 4^d^, 5^e^, 6^f^, 7^g^, 8^h^, 9^i^)
1	Bant	2.5	183	0.00	1, 5, 7
2	Best Diabetes Control	1	2	0.99 (Lite version available)	1, 2, 6, 9
3	Blood Diary	3	1	0.00	1, 2, 9
4	Blood Glucose Tracker (japps)	3.5	2	In-app purchases	1, 2, 3, 5, 9
5	Blood Sugar Diabetes Control	2.5	150	0.99	1, 2, 5, 6, 7
6	Dafne Online	0	0	0.00	1, 2, 5, 8
7	Dbees	3	16	0.00	1, 2, 5, 6, 9
8	Dblog	3	2	0.99	1, 7, 9
9	Diabetes 360	4	21	4.99	1, 2, 5, 7, 9
10	Diabetes App	4	2940	6.99	1, 2, 4, 5, 6, 7, 8, 9
11	Diabetes Assistant	4.5	5	1.99	1, 2, 3
12	Diabetes Companion	0	0	0.00	1
13	Diabetes Connect	4.5	67	In-app purchases	1, 2, 5, 7, 8, 9
14	Diabetes Diary	3.5	41	2.99	1, 2, 5, 9
15	Diabetes Factors	1	5	0.00	1, 5, 7, 8, 9
16	Diabetes Glucose Tracker app	2	85	2.99	1, 2, 3, 5, 7, 9
17	Diabetes Health Mate	4	17	0.00	1, 2, 3, 5, 9
18	Diabetes in Check	4	989	0.00	1, 2, 3, 4, 7, 9
19	Diabetes Kit	5	287	0.00	1, 2, 3, 5, 9
20	Diabetes Log	3	1948	0.00	1, 2, 7, 8, 9
21	Diabetes Logbook by mySugr	5	1684	In-app purchases	1, 2, 3, 5, 8, 9
22	Diabetes Logger	1	1	0.00	1, 2, 5, 9
23	Diabetes Management app	1	1	In-app purchases	1, 2, 7, 9
24	Diabetes Manager	5	2	4.99	1, 2, 5, 7, 9
25	Diabetes Pal app	4	183	0.00	1, 2, 5, 8, 9
26	Diabetes Parent	0	0	0.00	1, 2, 8, 9
27	Diabetes Passport	0	0	0.00	1, 2, 5, 7, 9
28	Diabetes Pilot Classic	4	221	24.99	1, 2, 4, 5, 7, 9
29	Diabetes Plus	4.5	8	3.99	1, 2, 5, 6, 7, 9
30	Diabetes Studio	0	0	0.00	1, 3, 5, 7, 8, 9
31	Diabetes Tracker with Blood Glucose/Carb Log by MyNetDiary	4.5	447	9.99	1, 2, 4, 5, 8, 9
32	Diabetes UK Tracker	0	0	0.00	1, 2
33	Diabetespal	4.5	6	2.99	1, 2, 5, 7, 9
34	Diabetesscs	0	0	0.00	1
35	Diabetesteam	0	0	0.00	1, 2, 5, 6, 7, 9
36	Diabetic Plus	0	0	0.00	1, 2, 3, 5, 6, 7, 9
37	Diabetic Plus	0	0	0.00	1, 2, 3, 5, 6, 7, 9
38	Diabetic Tracker Unlimited	2	25	1.99	1, 5, 7, 9
39	Diabeticplus	0	0	0.00	1, 5, 7, 8, 9
41	Diabetics Diary	4	1	0.00	1, 2, 6, 7, 9
42	Diabetic’s Diary	5	1	0.00	1, 2, 5, 6, 7, 9
43	Diabetik	5	79	0.00	1, 2, 3, 7, 9
44	Diabettes	0	0	0.00	1, 2, 6, 9
45	Diamedic	3.5	143	5.99	1, 2, 3, 5, 7, 9
46	Ditto Glucose Logbook	2	2	0.00	1, 2, 5, 7, 9
47	Dmdiary	5	1	0.00	1, 5, 7, 9
48	Easy Diabetes	4	29	0.00	1, 2, 5, 7, 9
49	Gestational Diabetes Manager	2.5	18	2.99	1, 2, 4, 6, 7
50	Glicontrol	1	1	In-app purchases	1, 2, 5, 7, 9
51	Glucocheck	0	0	0.00	1, 2, 5, 6, 9
52	GluCoMo	2	82	0.99	1, 3, 5, 9
53	Glucorecord	2	14	In-app purchases	1, 2, 5, 6, 7, 9
54	Glucose Buddy	4	6400	In-app purchases	1, 2, 3, 5, 7, 8, 9
55	Glucose Companion	4.5	888	1.99	1, 2, 3, 5, 7, 8, 9
56	Glucose Monsters	2	11	0.00	1, 2, 5
57	Glucose Readings	0	0	0.99	1, 5, 7, 9
58	Glucose Recorder	3.5	30	2.99	1, 2, 5, 8, 9
59	Glucose Tracker	2.5	17	1.99	1, 2, 5, 7, 9
60	Glucose Tracker - simple and complete app	5	6	0.99	1, 2, 3, 5, 7, 9
61	Glucose Wiz/Pro	4	61	1.99	1, 2, 3, 5, 7, 8, 9
63	Glucosurfer Free	0	0	0.00	1, 2, 5, 9
64	Glucosurfer	0	0	0.99	1, 2, 6, 7, 9
65	Glucosweet	2.5	7	6.99	1, 2, 5, 7, 8, 9
66	Glycemiaquicklog	2	1	2.99	1, 3, 5, 7, 9
67	Gmate	4	9	0.00	1, 2, 5, 6, 9
68	Healthediabetes	3.5	15	5.99	1, 2, 9
69	Glucose Monitor (HealthstomeG)	4.5	331	In-app purchases	1, 2, 3, 6, 7, 8, 9
70	Ibgstar Diabetes Manager	3.5	150	0.00	1, 2, 3, 5, 9
71	Iglu-bz	0	0	0.00	1, 2, 5, 6
72	Mdiabetes	0	0	0.99	1, 2, 4, 5, 7, 9
73	Mydiabetes	0	0	3.99	1, 2, 3, 5, 6, 7, 9
74	Mydiabetesapp	0	0	3.99	1, 2, 5, 6, 7, 9
75	Mysugr Junior	4	11	0.00	1, 2, 4, 7, 9
76	Onsync Diabetes Manager	2	28	0.00	1, 2, 5, 7, 9
77	Pomihealth	4.5	70	2.99	1, 2, 5, 9
78	Predict Bgl	5	3	0.00	1, 2, 4, 6, 9
79	Rapidcalc	4.5	25	7.99	1, 2, 5, 7, 9
80	Sidiary	3	9	5.99	1, 5, 8, 9
81	Simple Diabetes	0	0	0.00	1, 2, 7, 9
82	Sugar Sense	4.5	86	0.00	1, 2, 6, 7, 9
83	Sugarpal Diabetes Manager	3	1	3.99	1, 2, 5, 7, 9
84	Track3	4.5	820	5.99	1, 2, 6, 7, 8, 9
85	Your Diabetes Diary	4	1	0.00	1, 2, 5, 6, 7, 9

^a^Logs glucose levels.

^b^Logs water and carbohydrate intake, weight, body mass index, medication, and blood pressure.

^c^Reminders or push notifications.

^d^Food database.

^e^Charts.

^f^Exercise management.

^g^Email.

^h^Sync between devices.

^i^Miscellaneous (Twitter, password protection, retina display, barcode scanner, apple watch functionality, cloud syncing, and miscellaneous functions).

**Figure 1:**
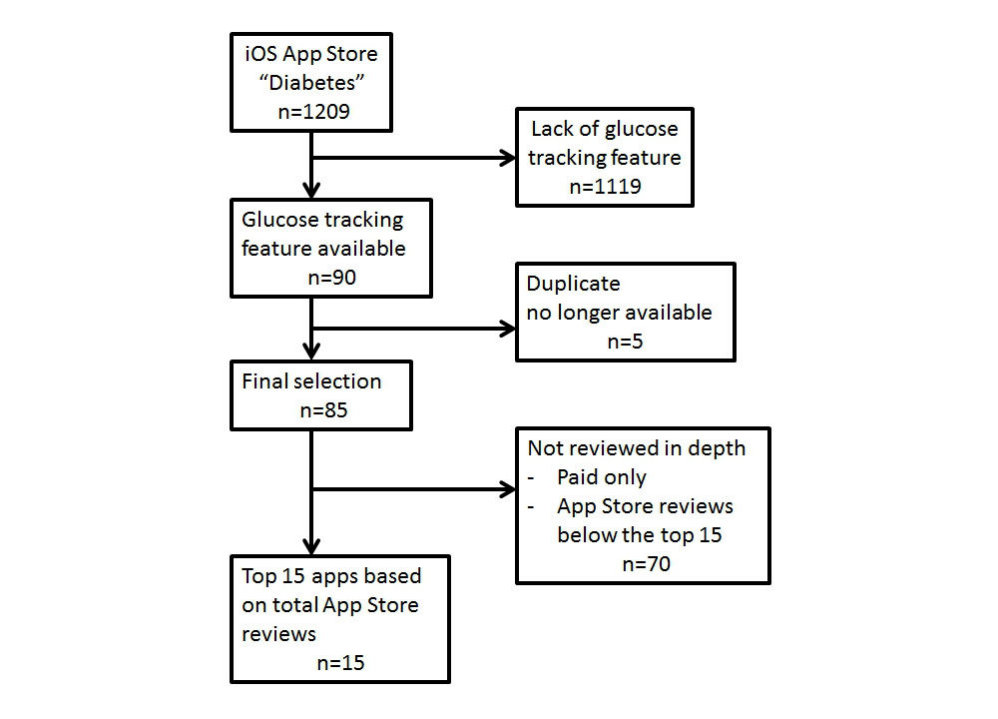
Selection algorithm of the top iOS apps for diabetes self-management.

Of these 1209 results, 85 were ultimately retained due to the presence of the ability to track users’ glucose levels (Table 1). The 1124 apps excluded focused mainly on diet, exercise, emergency services, providing general diabetes information, and other nontherapeutic options, or they were duplicate apps (Figure 1). The glucose levels tracked were visible on most apps from the landing page description. Any apps in which this information was not on the landing page were found using an iPhone, searching by the name of the app so as not to interrupt the search on the iPad. As performing the same “diabetes” search at different times or on different devices (iPhones) would result in varying results (different order or quantity), for the purpose of this analysis, we used the results for the search term “diabetes” from a single device (an iPad), and the author continuously reviewed the description of all the apps without interruption (this task required about six hours to accomplish). This method ensured that the list of search results would maintain the same apps in the same order. Based on these factors, our study focused on apps available in the iOS App Store.

### Apps Reviews and Analysis

After the inclusion criteria for the presence of glucose level tracking feature were met and the 85 apps were selected, the breakdown and recording of specific features for the respective apps was split amongst three of the authors (MM, EP, and IM). Information not available on the landing page was gathered by entering the specific app’s page on the iPad and reviewing the features. Each person recorded the rating for the app, number of reviews, price, in-app purchase facility, features, and major pros and cons noted in user reviews.

An in-depth analysis was then performed by two authors (MM and LS) on the “Top 15” apps, based on the highest number of reviews, and the information is presented below and in Multimedia Appendix 1. The respective apps were downloaded on iPhones and used over the course of 2 weeks, with each author performing a qualitative analysis of the apps’ ability to track glucose levels, carbohydrate intake, medication, weight, exercise, and blood pressure, as well as the apps’ ease of use and graphs. The availability of additional features such as data export, back up, goal setting, forums, and integration with a meter were also recorded. Each author allotted a rating of good or poor to the qualitative features. A good rating was given if the feature demonstrated a majority of the following characteristics: being intuitive and useful, stable (the app does not crash if the feature is used), and well designed in terms of colors, font, clarity, and easily accessible from the menu. At the end of the 2-week trial period, the two authors independently completed their comprehensive reviews of the 15 apps and then came together to resolve any discrepancies in the ratings and finalize the results.

## Results

Among the top 15 apps tested, only 13% (2/15) featured integration with a meter (Multimedia Appendix 1). Only 13% (2/15) featured the ability to receive advice through a certified diabetes educator within the app. Availability of tracking features was 73% (11/15), 73% (11/15), 73% (11/15), 53% (8/15), and 46% (7/15) for tracking carbohydrate intake, medication tracking, weight tracking, exercise tracking, and blood pressure tracking, respectively. Despite 73% (11/15) of the apps having the feature of tracking carbohydrate intake, only 3 of the 15 apps had an integrated food database; most of the apps focused on the input of carbohydrate values as opposed to the input of food with carbohydrate values calculated by the app itself. Less than half of the apps featured the availability of adherence reminders, and among these, three did not offer the feature in the free version. We found that 93% (14/15) of the apps featured a method to export data, primarily by emailing values or graphs, and 73% (11/15) of the apps allowed the user to set goals in order to visualize when they failed to meet their goals, generally using a certain color to indicate hypoglycemia or hyperglycemia. Seven apps featured some form of advertisement within the app that could be removed by upgrading from the free to the paid version. Only one app (SugarSense) mentioned the use of guidelines and provided users with information citing the guidelines, while also providing a link directly to the American Diabetes Association (ADA) guidelines.

## Discussion

### Principal Findings

Patient self-management of chronic illness is important to assist physicians in management of the disease and increase adherence. The use of apps has been shown to be useful not only in diabetes, but also in other chronic diseases such as cancer [26] as well as non-chronic diseases such as weight loss [27]. Data obtained from apps on blood glucose, blood pressure, diet, exercise, asthma exacerbations, and so forth could be instrumental in maintaining proper medication regimens and improving the effectiveness of targeted counseling from physicians, based on where the patient is failing or succeeding.

Subsequent to the review and comparison of the top 15 apps for tracking glucose levels, the primary differentiating factors among the apps were found to be their respective supplemental features such as carbohydrate-intake tracking, medication tracking, weight tracking, exercise tracking, blood pressure tracking, ease of use, food database, graph availability, adherence reminders, data export, data backup, goal setting, notes, advertisements, community forums, access to certified diabetes educators, and integration with a meter.

Our results reflected that patient reviews emphasized the desire for simplicity, but also the availability of more complex features (highly customizable graphs, data backup, and synchronization across devices). The best apps had a large number of features but did not overwhelm the user by displaying all of the features or customization options available. In the future, syncing data directly to prescribers could provide the patients’ detailed blood glucose readings, medication adherence practices, and diet in a standardized format. The data could potentially increase health care outcomes by providing a larger pool of data to improve pharmacologic therapy and non-pharmacologic therapy counseling for patients.

Although all of the apps reviewed were for diabetes management, less than half of the top apps (7/15) had a medication adherence function (Multimedia Appendix 1). It was puzzling that although so many apps had a comprehensive list of features that ranged from tracking calories to cloud backup, they failed to implement reminders for medication, as forgetfulness is a factor of nonadherence. This may have been because many patients inject per sliding scale or with meals and the timing is nontraditional; nevertheless, reminders should be a requirement of self-management apps. One stand out feature only apparent in the Diabetes Logbook app by mySugr was the introduction of fun as a method of increasing adherence. The highest rated apps had myriad features and many comparable features between them, but having “fun” while inputting data may add the extra push that users need to continue to use the app and attain their therapeutic goals.

### Limitations

There is neither regulatory body assigned to monitor the efficacy of wellness apps, nor a designated evidence base [28]. SugarSense was the only app in the top 15 that provided referenced information per ADA guidelines as well as a direct link to the ADA guidelines. Although this is important, usefulness for a patient who may be a layperson has to be evaluated. Kirwan et al have shown that an app supplemented with certified educator feedback via text messaging produced statistically significant improvement in the control of patients’ blood glucose levels [29]. While text-message feedback was not incorporated in our review, two of the top 15 apps (Diabetes Logbook and Diabetes Kit) did provide the optional resource of a certified diabetes educator. User reviews reflected mixed positive and negative opinions regarding the feature. Further evaluation is needed regarding degree of usefulness.

While there are a medley of apps available for assistance in self-management of diabetes and other chronic diseases, it is important to determine exactly which features are instrumental to the success of patient goals in disease management. Patients are easily discouraged by an abundance of features, but they are equally discouraged by a lack of features and customization. The exact components of an ideal self-management app may already be possessed by the apps discussed here; it is just a matter of optimization by the removal of unnecessary features and the addition of missing features.

This study was limited in terms of the time when the apps were introduced to the market, as this may have impacted the number of reviews—apps that were available for a longer period of time for user download and use may have had reviews that were more positive or numerous. This study may have also yielded different results had we also included the paid only apps in the top 15 list (Diabetes Pilot Classic, Blood Sugar Diabetes Control, Diabetes Diary, Diamedic, and GluCoMo).

### Conclusions

Apps may assist health care providers in inching closer to optimal prescriptions by increasing both patient involvement and availability of data. The use of phone apps for management of chronic diseases such as diabetes is not a novel concept, but the extent to which specific features may improve adherence along with real-world application by physicians has been minimally explored [25,30-35]. Features that should be considered by app developers are graph customization, availability of data backup, records of previous entries, and syncing directly from glucometers without the need for manual input of values. Two apps allowed syncing of glucose values directly from patient glucometers, which should increase ease of use. Ibgstar Diabetes Manager by Sanofi connects the glucometer directly to an iPhone or iPod touch and inputs glucose readings into the app. The Diabetes Pal App by Telcare provides similar functions and integration, but data from the glucometer is instead sent over wi-fi directly to the Telcare website, after which it can be synced to the app. The Telcare device was well received, whereas the Sanofi meter received a number of reviews complaining about lack of functions. Technology can assist in increasing patient compliance and quality of life in managing chronic illness; however, it is important for app developers to realize that ease, feasibility of customization, and number of functions is important for patient reliance, though developers should not get carried away and deviate from the original goal of working with patients and prescribers in order to improve health outcomes.

## Appendix

Multimedia Appendix 1 Attributes of the top 15 apps for diabetes self-management available in the iOS App Store.

## Abbreviations

T1DM: type 1 diabetes mellitus
T2DM: type 2 diabetes mellitus
BMI: body mass index
BP: blood pressure

## References

[1] Ward BW, Schiller JS, Goodman RA (2014). Multiple chronic conditions among US adults: a 2012 update. Prev Chronic Dis.

[2] Yun S, Kayani N, Homan S, Li J, Pashi A, McBride D, Wilson J (2013). The burden of chronic diseases in Missouri: progress and challenges. Mo Med.

[3] (2014). NIDDK.

[4] Mokdad AH, Ford ES, Bowman BA, Dietz WH, Vinicor F, Bales VS, Marks JS (2003). Prevalence of obesity, diabetes, and obesity-related health risk factors, 2001. JAMA.

[5] Nguyen NT, Nguyen XM, Lane J, Wang P (2011). Relationship between obesity and diabetes in a US adult population: findings from the National Health and Nutrition Examination Survey, 1999-2006. Obes Surg.

[6] Scott RA, Langenberg C, Sharp SJ, Franks PW, Rolandsson O, Drogan D, van der Schouw YT, Ekelund U, Kerrison ND, Ardanaz E, Arriola L, Balkau B, Barricarte A, Barroso I, Bendinelli B, Beulens JW, Boeing H, de Lauzon-Guillain B, Deloukas P, Fagherazzi G, Gonzalez C, Griffin SJ, Groop LC, Halkjaer J, Huerta JM, Kaaks R, Khaw KT, Krogh V, Nilsson PM, Norat T, Overvad K, Panico S, Rodriguez-Suarez L, Romaguera D, Romieu I, Sacerdote C, Sánchez MJ, Spijkerman AM, Teucher B, Tjonneland A, Tumino R, van der A DL, Wark PA, McCarthy MI, Riboli E, Wareham NJ, InterAct Consortium (2013). The link between family history and risk of type 2 diabetes is not explained by anthropometric, lifestyle or genetic risk factors: the EPIC-InterAct study. Diabetologia.

[7] Reis JP, Loria CM, Sorlie PD, Park Y, Hollenbeck A, Schatzkin A (2011). Lifestyle factors and risk for new-onset diabetes: a population-based cohort study. Ann Intern Med.

[8] Shai I, Jiang R, Manson JE, Stampfer MJ, Willett WC, Colditz GA, Hu FB (2006). Ethnicity, obesity, and risk of type 2 diabetes in women: a 20-year follow-up study. Diabetes Care.

[9] (2014). CDC.

[10] (2014). CDC.

[11] Garber AJ, Abrahamson MJ, Barzilay JI, Blonde L, Bloomgarden ZT, Bush MA, Dagogo-Jack S, DeFronzo RA, Einhorn D, Fonseca VA, Garber JR, Garvey WT, Grunberger G, Handelsman Y, Henry RR, Hirsch IB, Jellinger PS, McGill JB, Mechanick JI, Rosenblit PD, Umpierrez GE, American Association of Clinical Endocrinologists (AACE), American College of Endocrinology (ACE) (2016). CONSENSUS STATEMENT BY THE AMERICAN ASSOCIATION OF CLINICAL ENDOCRINOLOGISTS AND AMERICAN COLLEGE OF ENDOCRINOLOGY ON THE COMPREHENSIVE TYPE 2 DIABETES MANAGEMENT ALGORITHM--2016 EXECUTIVE SUMMARY. Endocr Pract.

[12] Bennett WL, Maruthur NM, Singh S, Segal JB, Wilson LM, Chatterjee R, Marinopoulos SS, Puhan MA, Ranasinghe P, Block L, Nicholson WK, Hutfless S, Bass EB, Bolen S (2011). Comparative effectiveness and safety of medications for type 2 diabetes: an update including new drugs and 2-drug combinations. Ann Intern Med.

[13] American Diabetes Association (2014). Standards of medical care in diabetes--2014. Diabetes Care.

[14] Haynes RB, McDonald H, Garg AX, Montague P (2002). Interventions for helping patients to follow prescriptions for medications. Cochrane Database Syst Rev.

[15] (2009). NEHI.

[16] Osterberg L, Blaschke T (2005). Adherence to medication. N Engl J Med.

[17] Miller KM, Beck RW, Bergenstal RM, Goland RS, Haller MJ, McGill JB, Rodriguez H, Simmons JH, Hirsch IB, T1D Exchange Clinic Network (2013). Evidence of a strong association between frequency of self-monitoring of blood glucose and hemoglobin A1c levels in T1D exchange clinic registry participants. Diabetes Care.

[18] Nadkarni A, Kucukarslan SN, Bagozzi RP, Yates JF, Erickson SR (2010). A simple and promising tool to improve self-monitoring of blood glucose in patients with diabetes. Diabetes Res Clin Pract.

[19] Lane SJ, Heddle NM, Arnold E, Walker I (2006). A review of randomized controlled trials comparing the effectiveness of hand held computers with paper methods for data collection. BMC Med Inform Decis Mak.

[20] Anderson M (2015). Pew Internet.

[21] El-Gayar O, Timsina P, Nawar N, Eid W (2013). Mobile applications for diabetes self-management: status and potential. J Diabetes Sci Technol.

[22] Arnhold M, Quade M, Kirch W (2014). Mobile applications for diabetics: a systematic review and expert-based usability evaluation considering the special requirements of diabetes patients age 50 years or older. J Med Internet Res.

[23] Eng DS, Lee JM (2013). The promise and peril of mobile health applications for diabetes and endocrinology. Pediatr Diabetes.

[24] Tran J, Tran R, White JR (2012). Smartphone-based glucose monitors and applications in the management of diabetes: an overview of 10 salient “apps” and a novel smartphone-connected blood glucose monitor. Clin Diabetes.

[25] Demidowich AP, Lu K, Tamler R, Bloomgarden Z (2012). An evaluation of diabetes self-management applications for Android smartphones. J Telemed Telecare.

[26] Wesley KM, Fizur PJ (2015). A review of mobile applications to help adolescent and young adult cancer patients. Adolesc Health Med Ther.

[27] Chin SO, Keum C, Woo J, Park J, Choi HJ, Woo JT, Rhee SY (2016). Successful weight reduction and maintenance by using a smartphone application in those with overweight and obesity. Sci Rep.

[28] Ozdalga E, Ozdalga A, Ahuja N (2012). The smartphone in medicine: a review of current and potential use among physicians and students. J Med Internet Res.

[29] Kirwan M, Vandelanotte C, Fenning A, Duncan MJ (2013). Diabetes self-management smartphone application for adults with type 1 diabetes: randomized controlled trial. J Med Internet Res.

[30] Bloss CS, Wineinger NE, Peters M, Boeldt DL, Ariniello L, Kim JY, Sheard J, Komatireddy R, Barrett P, Topol EJ (2016). A prospective randomized trial examining health care utilization in individuals using multiple smartphone-enabled biosensors. PeerJ.

[31] Cafazzo JA, Casselman M, Hamming N, Katzman DK, Palmert MR (2012). Design of an mHealth app for the self-management of adolescent type 1 diabetes: a pilot study. J Med Internet Res.

[32] Coughlin SS, Whitehead M, Sheats JQ, Mastromonico J, Hardy D, Smith SA (2015). Smartphone applications for promoting healthy diet and nutrition: a literature review. Jacobs J Food Nutr.

[33] Dayer L, Heldenbrand S, Anderson P, Gubbins PO, Martin BC (2013). Smartphone medication adherence apps: potential benefits to patients and providers: response to Aungst. J Am Pharm Assoc (2003).

[34] Hale K, Capra S, Bauer J (2015). A framework to assist health professionals in recommending high-quality apps for supporting chronic disease self-management: illustrative assessment of type 2 diabetes apps. JMIR Mhealth Uhealth.

[35] Rao A, Hou P, Golnik T, Flaherty J, Vu S (2010). Evolution of data management tools for managing self-monitoring of blood glucose results: a survey of iPhone applications. J Diabetes Sci Technol.

