# Supplementary figures and images for: iOS Appstore-Based Phone Apps for Diabetes Management: Potential for Use in Medication Adherence
Source: JMIR Diabetes. 2017 Jul 11;2(2):e12. doi: 10.2196/diabetes.6468 (PMC6238890; doi:10.2196/diabetes.6468)

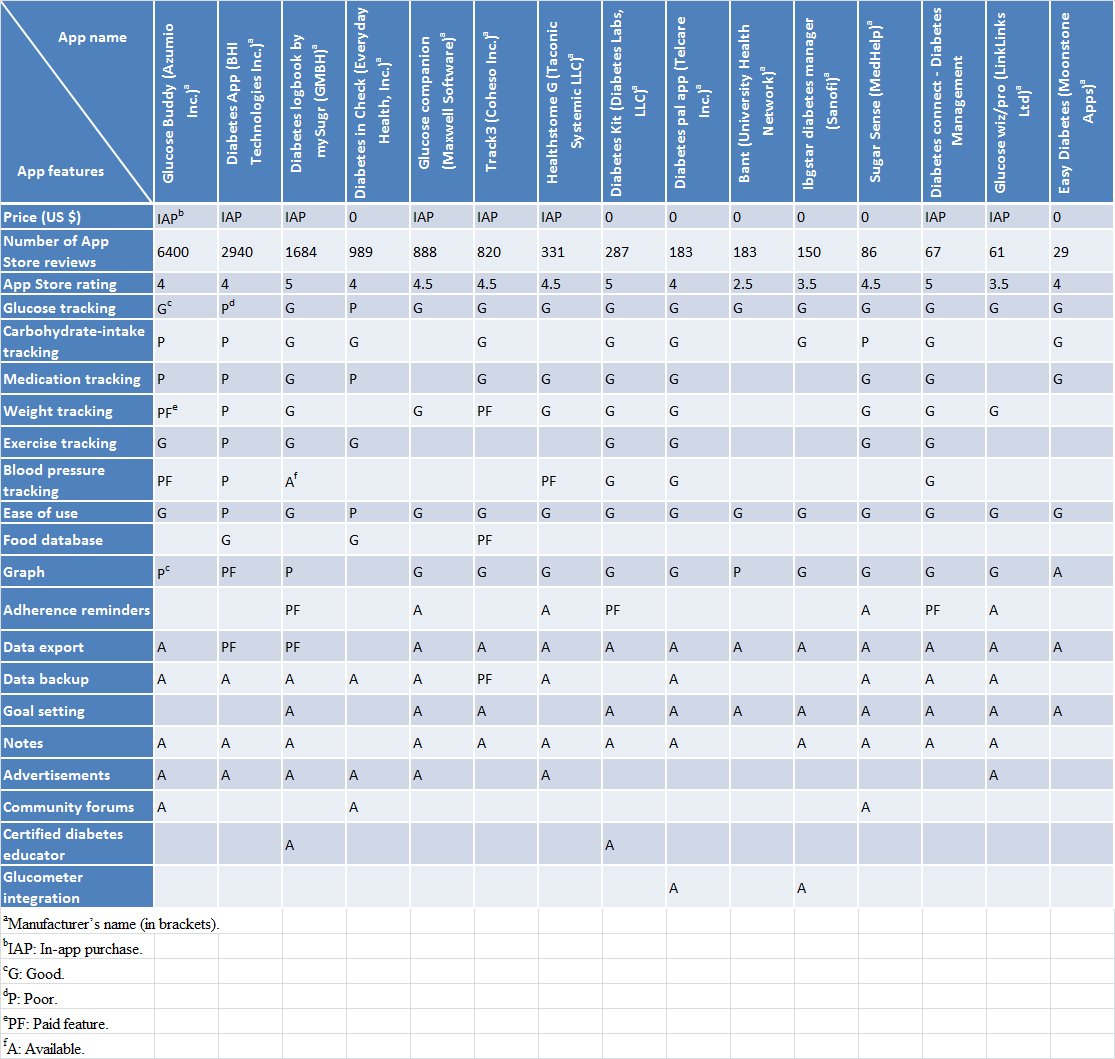

Supplement: Multimedia Appendix 1 [file diabetes_v2i2e12_app1.jpg]
